# Nano-Based Drug Approaches to Proliferative Vitreoretinopathy Instead of Standard Vitreoretinal Surgery

**DOI:** 10.3390/ijms25168720

**Published:** 2024-08-09

**Authors:** Francesco Saverio Sorrentino, Lorenzo Gardini, Carola Culiersi, Luigi Fontana, Mutali Musa, Fabiana D’Esposito, Pier Luigi Surico, Caterina Gagliano, Marco Zeppieri

**Affiliations:** 1 Department of Surgical Sciences, Unit of Ophthalmology, Ospedale Maggiore, 40100 Bologna, Italy; dr.fsorrentino@gmail.com (F.S.S.);; 2 Department of Surgical Sciences, Ophthalmology Unit, Alma Mater Studiorum University of Bologna, IRCCS Azienda Ospedaliero-Universitaria Bologna, 40100 Bologna, Italy; 3 Department of Optometry, University of Benin, Benin City 300238, Edo State, Nigeria; 4 Africa Eye Laser Centre, Km 7, Benin 300105, Nigeria; 5 Imperial College Ophthalmic Research Group (ICORG) Unit, Imperial College, 153-173 Marylebone Rd., London NW15QH, UK; 6 Department of Neurosciences, Reproductive Sciences and Dentistry, University of Naples Federico II, Via Pansini 5, 80131 Napoli, Italy; 7 Schepens Eye Research Institute of Mass Eye and Ear, Harvard Medical School, Boston, MA 02114, USA; 8 Department of Ophthalmology, Campus Bio-Medico University, 00128 Rome, Italy; 9 Department of Medicine and Surgery, University of Enna “Kore”, Piazza dell’Università, 94100 Enna, Italy; 10 Eye Clinic, Catania University, San Marco Hospital, Viale Carlo Azeglio Ciampi, 95121 Catania, Italy; 11 Department of Ophthalmology, University Hospital of Udine, 33100 Udine, Italy

**Keywords:** nanomedicine, drug-delivery systems, ocular nanomedicine, nanotechnologies, targeted therapy, tailored therapy, vitreoretinal diseases

## Abstract

Proliferative vitreoretinopathy (PVR) has traditionally been managed with vitreoretinal surgery. Although there have been several recent innovations in this surgery to make the retinal approach as uninvasive as possible, the outcomes remain unsatisfactory. Significant complications remain and the complexity of the surgical approach is challenging. The focus of this review was to investigate and discuss the effectiveness of nanomedicine, featuring a wide range of drugs and molecules, as a novel potential treatment for PVR. To date, ocular drug delivery remains a significant issue due to the physiological and anatomical barriers, dynamic or static, which prevent the entry of exogenous molecules. We tried to summarize the nanotechnology-based ophthalmic drugs and new nanoparticles currently under research, with the intention of tackling the onset and development of PVR. The purpose of this review was to thoroughly and analytically examine and assess the potential of nano-based techniques as innovative strategies to treat proliferative vitreoretinopathy (PVR). This study aimed to emphasize the breakthroughs in nanomedicine that provide promising therapeutic options to enhance the results of vitreoretinal surgery and halt disease progression, considering the complexity and difficulty of PVR treatment. The future directions of the nanoparticles and nanotherapies applied to PVR highlight the importance of investing in the development of better designs and novel ophthalmic formulations in order to accomplish a mini-invasive ocular approach, replacing the standard-of-care vitreoretinal surgery.

## 1. Introduction

Nanomedicine has made significant progress in treating many eye illnesses such as PVR, expanding the possibilities for their therapy. Nanotechnology-enabled drug-delivery technologies such as nanoparticles, liposomes, and dendrimers provide precise delivery, regulated release, and improved availability of medicinal drugs. Nano-based strategies have demonstrated promise in surmounting the biological obstacles of the eye, diminishing adverse reactions, and enhancing patient adherence.

This study includes a comprehensive explanation of the underlying mechanisms of PVR, followed by a detailed examination of the most recent advancements in nano-based treatments. We analyze the potential integration of these innovative methods into existing treatment frameworks to optimize the effectiveness and safety of PVR control, with the ultimate goal of enhancing patients’ visual results.

PVR is a multifactorial process featuring the presence of many different cells (macrophage, glial, and RPE cells) and cytokines, bringing about profibrotic and proinflammatory activity [1,2]. Its main feature is the pooling of epiretinal and subretinal fibrocellular membranes, which results in fibrosis on both sides of the retinal surface. The contraction and shrinking of these membranes provoke the recurrence of RD and a rhegmatogenous detachment changes into a tractional detachment within the vitreous cavity [3]. The epi/intraretinal fibrosis modifies the multilayered retinal tissue, preventing the retina from flattening even after pulling off all membranes.

The PVR incidence in all cases of RD is roughly estimated to be 5–10% [4]. In spite of the evolution of vitreoretinal techniques over the past 20 years, including valved trocars and smaller gauge instrumentation, the PVR incidence has largely remained constant [5,6]. This is the point to better understand the pathogenic process of PVR and to identify the key molecules involved in PVR early onset in order to develop nanotherapies with specific targets and to try to prevent a fibrotic retinal response after vitreoretinal surgery [7].

This mini-review examines the pathogenesis of subretinal, intraretinal, and epiretinal PVR and briefly discusses the current pharmacological strategies compared with the classic surgical approach of vitrectomy.

## 2. Methods

In order to compile this review, a thorough search of the literature was performed to find pertinent papers about medication approaches based on nanotechnology for proliferative vitreoretinopathy (PVR). Scopus, Web of Science, and PubMed were among the databases that were searched. Keywords included “ocular drug delivery”, “proliferative vitreoretinopathy”, “PVR”, “nanomedicine”, “nanoparticles”, “vitreoretinal surgery”, “inflammation”, and “fibrosis”. Articles were chosen based on research concentrating on PVR therapy with nanotechnology; peer-reviewed publications that published research articles, reviews, and clinical trials; research addressing PVR pathogenesis in connection with nanomedicine; and English-language articles. The exclusion criteria were based on those studies having nothing to do with PVR or nanotechnology, research with only an emphasis on surgical methods, conference proceedings, abstracts, and non-peer-reviewed articles.

The search approach using general keywords produced a large number of papers about PVR and nanotechnology. Relevance was determined by looking through the titles and abstracts of the identified publications. The full texts of the shortlisted articles were examined to make sure they satisfied the requirements for inclusion. To find further pertinent research, the reference lists of a few chosen papers were examined. The assessment of articles included relevance or the extent to which PVR applications of nanomedicine were covered in the study. The study’s methodological rigor was evaluated by taking into account the statistical analysis, experimental design, and sample size. The method’s originality and possible influence on PVR treatment were also considered.

The information that was taken from the chosen publications had an emphasis on nanoparticle types (i.e., mesoporous silica nanoparticles, metallic nanoparticles, polymeric nanoparticles, and lipid-based nanoparticles); mechanisms of action; preclinical and clinical findings; safety, effectiveness, and possible adverse effects noted in research involving humans and animal models; and future directions and recommendations for enhancements.

An extensive summary of the state of nanotechnology related to PVR treatment was produced by synthesizing the retrieved data. The synthesis centered on a comparative analysis based on evaluating the efficacy of various nanoparticle forms. The assessment provided information about the integration with current therapies and how surgical procedures can be supplemented by nanomedicine. Determining the principal roadblocks to the creation and clinical implementation of nanotechnology-based treatments was also assessed. The current literature also provided information about the potential of clinical translation by evaluating these treatments’ suitability for clinical trials and potential therapeutic applications.

## 3. Pathogenesis of Proliferative Vitreoretinopathy

The pathogenesis of proliferative vitreoretinopathy (PVR) is intricate and not entirely understood, involving diverse cells—for instance, retinal pigment epithelial (RPE), glial, fibroblast, and many inflammatory cells [8]—all up- and downregulated by growth factors, inflammatory signals, and cytokines [9]. Distinct pathways of cell death such as apoptosis and necrosis are present.

**Figure 1:**
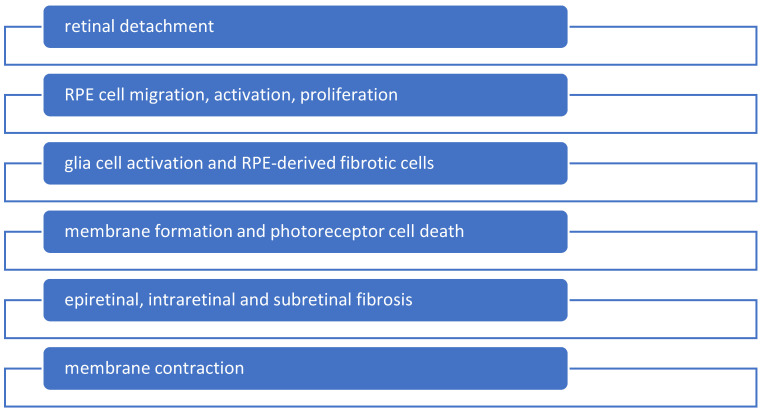
Due to retinal detachment, some molecular alterations take place in the microenvironment of the detached retina, such as the breakdown of the blood–retinal barrier, retinal hypoxia, the activation of glial and retinal pigment epithelium (RPE) cells, and subsequent inflammation. A range of chemokines and cytokines provoke RPE activation, which results in RPE-derived fibrotic cells that give birth to membrane formation.

Several types of cells play roles in retinal photoreceptor degeneration and may contribute to PVR development by affecting fibrosis formation during wound healing. Recent studies emphasize the significant role of Müller glia in PVR, showing that their response to pathogenic stimuli includes augmented proliferation, hypertrophy, and upregulation of fibrillary filaments such as vimentin, nestin, and glial fibrillary acidic protein (GFAP) [10]. Released by glial cells, signaling molecules regulate the microenvironment of microinflammation and local immunity, comprising chemotactic substances (MCP-1 and MIP-1α) and proinflammatory cytokines (IL-8, IL-6, and TNF-α) [11]. Toll-like receptors and MHC II molecules within the microenvironment of retinal detachment with oxidative stress have been observed in research models (Figure 1).

CXCL5 is a notable biomarker for the development of postoperative PVR [12]. RPE cells are major contributors to PVR, comprising 50–90% of cells in subretinal membranes from enucleated eye specimens. RPE cells release proteins such as PDGF, FGF, and TGF-β involved in autocrine growth regulation. RPE cells can also transdifferentiate into a mesenchymal phenotype, acquiring abilities to migrate, resist apoptosis, produce metalloproteases, and remodel the extracellular matrix. Macrophages are also crucial in PVR pathogenesis [13]. Their presence in the vitreous correlates with a higher risk of developing PVR. Macrophages not only exhibit proinflammatory activity but also mediate photoreceptor cell apoptosis through MCP-1. The interplay among different cell types, cytokines, and mediators in PVR creates a feedback loop, sustaining the disease [14]. This complex network of cellular interactions and signaling pathways complicates the development of effective treatments for PVR.

## 4. Surgical Treatment

Despite extensive research into medical therapies, surgery remains the primary treatment for most vitreoretinal surgeons to primarily further the PVR approach. The surgical technique is highly complex and not well-standardized, leaving much to the personal experience of the surgeon. Pars plana vitrectomy (PPV) is a crucial option for the management of PVR. Total vitreous removal, including the meticulous shaving of the base and peeling of all pre-retinal membranes, is essential [15]. The peeling of the ILM at the posterior pole is also advisable, reducing the recurrence of epiretinal membranes and the risk of re-detachment [16].

The timing of surgery is particularly contentious. If possible, it is recommended that surgery is delayed to allow the epiretinal membranes to mature as fibrotic membranes are easier to remove than fragile and immature ones. Nevertheless, if the macula is involved and vision is seriously affected, with visual outcomes at immediate risk, early intervention is absolutely recommended [17]. The presence of subretinal bands can variably affect retinal reattachment. If they are large, they can be removed by a small retinotomy or a wide retinectomy. Some authors suggest using a single bubble of PFCL in the subretinal space to fold the retina and improve visualization for a more accurate subretinal PVR removal [18]. However, in more severe cases of PVR, the presence of intraretinal fibrosis often requires a combined surgical approach with the placement of a circumferential scleral buckle for permanent retinal support followed by definitive vitreous surgery to relieve retinal traction [17]. Recent studies have demonstrated advances in the surgical management of RD with PVR, although the recurrence rate of the onset of PVR after RD continues to represent a crucial limitation to surgical success [2].

The use of retinotomy or retinectomy should be regarded as a last extreme surgical option if the retina cannot be properly flattened and reattached after the removal of all possible membranes. Retinotomy is effective in most cases, achieving initial anatomical success in 73.8% of eyes. However, some eyes require additional surgery for final success [19]. In the presence of widespread preoperative PVR with significant retinal stiffness that requires numerous retinectomies, Caporossi et al. recommended extensive coverage of the exposed RPE using human amniotic membrane (hAM) patches. These patches, made of biological components, appear to prevent further postoperative PVR by restoring contact inhibition and suppressing the TGF-β signaling pathway, with promising results in retinal reattachment and improved visual acuity [20].

Regarding the choice of tamponade to ensure retinal reattachment, the Silicone Study compared the effectiveness of different agents to manage PVR. It found that silicone oil and C3F8 were equally effective in anatomical and visual outcomes, and was superior to SF6 in visual outcomes [15]. Therefore, the tamponade agent selection should be tailored to each patient, considering factors such as intraoperative ocular findings and personal preference. Oxane-heavy silicone oil is sometimes preferred, especially for complicated inferior PVR and tears [21].

One of the latest technologies available during surgery is intraoperative OCT (I OCT), which plays a crucial role in visualizing retinal changes and facilitates the identification of subretinal membranes [22]. In such cases, the visual outcome is often very poor. The results obtained remain insufficient, particularly in terms of cost-effectiveness and patient satisfaction.

## 5. Nanotechnologies and Nanoparticles

Nanoparticles (NPs) are minuscule carriers capable of encapsulating medication, safeguarding them against degradation and enabling precise delivery to certain cells or tissues. PVR has been investigated using many types of nanoparticles. Lipid-based nanoparticles, including liposomes and solid lipid nanoparticles (SLNs), can encapsulate hydrophobic medicines, thus enhancing their solubility and durability. Additionally, they can be modified with ligands to improve their ability to specifically target certain cell types such as RPE cells, which are involved in PVR [23].

Biodegradable polymers such as PLGA (polylactic-co-glycolic acid) are frequently employed to produce nanoparticles that offer regulated medication release. These nanoparticles can be manipulated to gradually release substances that reduce inflammation and cell growth, thus decreasing the necessity for regular injections into the eye [24].

Metallic nanoparticles, specifically gold and silver NPs, have been studied for their ability to inhibit fibrosis and reduce inflammation. Additionally, these NPs can function as vehicles for medication or can be utilized alongside laser treatments to augment the effectiveness of the therapy [25].

Mesoporous silica nanoparticles (MSNs) possess a significant ability to carry a large amount of drugs and can be modified with different targeting molecules. They have demonstrated potential in delivering therapeutic substances directly to the location of fibrosis, thereby reducing the advancement of PVR [26].

Therapies that specifically target specific cells or molecules and agents that prevent or reduce the formation of fibrous tissue have been examined [27].

Precision in delivering medication is essential to reduce adverse effects and improve the effectiveness of PVR therapies. Nanotherapies can be engineered to selectively target the molecular pathways implicated in the development of PVR, including the TGF-β signaling pathway, which plays a crucial role in regulating fibrosis. By conjugating NPs with antibodies or peptides that specifically target these pathways, it becomes feasible to directly administer anti-fibrotic drugs to the cells that are impacted [28].

## 6. Pharmacological Therapy

Various novel agents are being investigated to improve PVR surgery outcomes. This review provides an overview of past and current investigational treatments and their potential clinical applications for the management of PVR. Currently, no specific nanotherapy has been approved as an adjunctive treatment to PVR surgery. Given the disease’s pathogenetic mechanisms, various drugs targeting inflammation, cell proliferation, and fibrosis have been explored, mainly in vitro, as illustrated in Table 1. Methotrexate, corticosteroids, and other anti-proliferative molecules have shown promising results in preclinical models and their clinical application is now being investigated.

### 6.1. Methotrexate

Methotrexate (MTX) is a folate antagonist with anti-proliferative and anti-inflammatory properties. It reduces DNA replication and cell proliferation while stimulating adenosine release. In vitro studies using human PVR membranes have shown that MTX can decrease RPE cell proliferation and migration, induce apoptosis, and avoid photoreceptor toxicity, unlike 5-fluorouracil [29]. Clinical studies have explored various MTX dosages (100 to 400 μg) and delivery methods, including single and multiple postoperative intravitreal injections and intraoperative infusions. Early studies reported promising results with a single intraoperative MTX injection during vitrectomy with silicone oil for grade C PVR [40]. In one series, an 80% success rate was noted after an average 25-month follow-up, with a BCVA increase from hand motion to a 20/200 median and only one case of superficial punctate keratopathy (SPK) [41]. Corneal epitheliopathy is the most common adverse event associated with intravitreal MTX, occurring in about 15% of patients, with the incidence reduced by extending the interval between injections. The preliminary results from the multicenter randomized phase III GUARD trial (NCT04136366) demonstrated some clinical benefits for an MTX 0.8%-treated group related to standard-of-care surgery. There was a complete retinal attachment, a lower RD rate, a low risk of hypotony, and reduced membrane formation after six months without functional distinctions or safety worries between the groups.

### 6.2. Daunomycin

Daunomycin is an anthracycline that inhibits cell proliferation and migration, and has shown effectiveness against PVR in animal models. A randomized multicenter trial (the Daunomycin Study Group) involving 286 grade C2 PVR patients evaluated the efficacy of an intraoperative daunomycin infusion compared with vitrectomy alone. After six months, 62.7% of the daunomycin group achieved complete retinal reattachment vs. 54.1% in the control group (*p* = 0.07). The daunomycin group also required fewer reoperations within a year to achieve the same reattachment rate (80.2% vs. 81.1%; *p* = 0.005). No significant differences in BCVA were observed, and daunomycin was well-tolerated. Despite these promising results, further clinical trials were not conducted due to non-significant primary outcomes, the challenges of using an anti-neoplastic agent in ophthalmology, and safety concerns [30].

### 6.3. Corticosteroids

Corticosteroids were the first agents studied for the management of PVR due to their broad anti-inflammatory and anti-proliferative properties, various administration methods, and lack of retinal toxicity evidence. Animal models have shown steroids’ efficacy in preventing or treating PVR. For example, in a rabbit model of PVR, triamcinolone acetonide at 2 mg reduced PVR-related RD incidence from 90% to 56% [42]. Another study found that intravitreal triamcinolone acetonide reduced RD incidence from 93% to 75% after 28 days and from 85% to 43% when prophylactically administered [43]. Despite these promising preclinical results, clinical studies have shown mixed outcomes. In a randomized clinical trial of 75 eyes with RD and grade C PVR undergoing vitrectomy with a silicone oil tamponade, adjunctive triamcinolone showed no significant differences compared with the controls [31]. Another study with 13 eyes reported mixed visual outcomes, but reported reattached retinas in 10 out of 13 eyes after nearly five months with no signs of re-proliferation in 8 eyes [32]. Koerner et al. found visual improvements and reattached retinas in 87.5% of 24 eyes with advanced grade C2 PVR after vitrectomy and an oil tamponade [44]. However, systemic steroid administration showed weaker effects on reducing fibrosis and improving visual outcomes compared with intravitreal triamcinolone acetonide. Sustained delivery systems maintaining active vitreous drug concentrations for longer showed promising preclinical results [45]. However, a two-year randomized prospective study with 140 patients using intraoperative dexamethasone implants showed no significant anatomical or functional improvements compared with the control group [46]. Steroids have also been studied in open globe trauma (OGT) cases undergoing vitrectomy. Although the initial results were promising, a phase 3 multicenter trial found no treatment benefits for adjunctive triamcinolone acetonide compared with standard care [47]. Additionally, a small trial combining triamcinolone with heparin during vitrectomy failed to show clinical benefits for established PVR [48].

### 6.4. Retinoic Acid

In vitro studies have revealed that retinoic acid inhibits the growth of RPE cells. A prospective randomized investigation evaluated the efficacy of 13-cis-retinoic acid (10 mg orally twice daily for 8 weeks postoperatively) in people with primary RD as well as PVR. Among 35 patients, 16 were treated with retinoic acid and 19 were in a placebo group. Retinal attachment was accomplished in 93.8% of the retinoic acid group vs. 63.2% in the controls (*p* = 0.047). Also, the retinoic acid group had a lower rate of macular pucker formation (18.8% vs. 78.9%; *p* = 0.001) and higher rates of visual function (56.3% vs. 10.5%; *p* = 0.009). The DELIVERY trial observed the effect of low-dose oral isotretinoin (20 mg daily for 12 weeks post-surgery) in 109 eyes, showing promising results in reducing the risk of PVR after RD surgery, particularly in high-risk cases. However, due to safety concerns, no further extensive studies have been conducted [33].

### 6.5. Low-Molecular-Weight Heparin (LMWH) and 5-Fluorouracil (5-FU)

Animal studies have shown that LMWH can reduce tractional RD rates and postoperative fibrin after vitrectomy. Additionally, 5-FU, an anti-neoplastic anti-metabolite, has proven effective in decreasing PVR rates in animal models. A randomized multicenter trial investigated a combination of 5-FU and LMWH in PVR prevention. Both the 5-FU/LMWH group and the placebo group had 87 patients each. The incidence of postoperative PVR was significantly lower in the 5-FU/LMWH group (12.6%) compared with the placebo group (26.4%; *p* = 0.02). Furthermore, fewer patients in the 5-FU/LMWH group required multiple procedures due to PVR compared with the placebo group. However, no significant difference in visual acuity was observed between the groups. A recent randomized, double-blinded, multicenter trial with 325 patients found no significant differences in PVR rates between 5-FU/LMWH and placebo groups [34].

### 6.6. Other Anti-Proliferative Molecules

Mitomycin C has shown potential in reducing post-traumatic PVR rates and improving anatomical and functional outcomes, but further randomized controlled studies are needed. Several other anti-proliferative molecules, including taxol, colchicine, glucosamine, alkyphosphocoline, and palomid, have shown promise in preclinical models, but no clinical trials have been conducted to assess their efficacy and safety. An ongoing phase II trial (NCT05523869) is investigating the intravitreal administration of topotecan for PVR-induced recurrent RD. A pilot study also suggested that a postoperative curcumin infusion might reduce PVR risk, but larger trials are required for confirmation [35].

### 6.7. Anti-VEGF Agents

Recent studies have highlighted the involvement of growth factors, particularly vascular endothelial growth factor (VEGF A), in the pathogenesis of proliferative vitreoretinopathy (PVR). VEGF A activates the platelet-derived growth factor (PDGF) receptor α, which plays a crucial role in PVR development. Animal studies have shown that ranibizumab, an anti-VEGF agent targeting all isoforms of VEGF-A, effectively reduces the bioactivity of the vitreous in experimental PVR and prevents PVR in rabbits. However, the clinical results have been less promising. A prospective study investigated the effects of serial intrasilicone oil injections of bevacizumab (1.25 mg/0.05 mL) on BCVA and anatomical success rates in patients with PVR-induced retinal detachment (RD). The study found no significant differences in the final BCVA, retinal reattachment rates, or epiretinal membrane formation between bevacizumab-treated patients and the controls. Similar outcomes were reported in another study. A meta-analysis of 133 studies concluded that intravitreal bevacizumab injections during vitrectomy for PVR-related RD were ineffective in reducing retinal re-detachment rates or improving visual acuity [36].

### 6.8. Other Targets under Early Investigation

#### 6.8.1. Extracellular Matrix

A new proteoglycan molecule called decorin inhibits TGF-β and extracellular matrix synthesis. Decorin was studied in a phase I trial to prevent PVR following ocular injuries. Patients were administered a single intravitreal dose of human recombinant decorin after the trauma, but before vitrectomy. There were no significant adverse effects at either the higher dose (400 μg) or the lower dose (200 μg). Further research is necessary to assess the efficacy of decorin [37].

#### 6.8.2. Platelet-Derived Growth Factor Receptor (PDGFR)

Regarding the pivotal role of PDGF and its receptor in the pathogenesis of PVR, targeting this pathway is not only a challenging but also a very promising strategy. A PDGFR kinase inhibitor, AG1295, was investigated in a rabbit model with PVR. In the treatment group, rabbits received weekly intravitreal injections of AG1295 for a total of four doses. The results pointed out that AG1295 diminished the development of PVR and reduced the incidence of tractional RD in rabbits. The drug seems to impact both RPE and glial cell proliferation without significant adverse effects [38].

#### 6.8.3. Epithelial-to-Mesenchymal Transition

MicroRNAs (miRNAs), particularly miR-194, contribute to RPE cell activating and migrating processes, and inhibit the epithelial–mesenchymal transition (EMT) phenotype. In a PVR rat model, the intravitreal administration of miR-194 broke down the development of PVR. Moreover, the protein kinase A (PKA) pathway has been investigated because it is involved in narrow interactions with TGF-β signaling and the EMT. The PKA inhibitor H89 was observed to lower the EMT and epiretinal membrane formation in both an in vitro and a PVR rat model [39].

#### 6.8.4. Combination Drug Delivery

Combination therapy targeting inflammatory and proliferative pathways has shown good potential outcomes. In one study, oxidized porous silicon particles loaded with dexamethasone and daunorubicin were administered into the vitreous cavity of a rabbit model affected by RD. This combination reduced the cellular markers of proliferation. However, caution is necessary as many animal models have responded well to pharmacological therapy in vitro, but human studies have not shown equally encouraging results.

Although several pharmacological agents have shown promise in preclinical studies, their translation into effective clinical treatments for PVR has been challenging. The intricate network of cellular interactions and signaling pathways in PVR complicates the development of effective nanotherapies. Further research and well-designed clinical trials are needed to identify safe and effective pharmacological adjuncts to surgery for PVR management.

## 7. Future Directions of Nanotherapies

The innovation of nanotechnologies and nanotherapies to treat PVR remains challenging. There are some problematic issues regarding the actual efficacy of these nanotherapies and their successful application in vivo to manage retinal diseases and their complications, such as PVR formation. Below, we discuss some aspects of the revolutionary modalities of precision nanomedicine and personalized nanotherapies.

Nanoparticles (NPs) facilitate the transport of genetic material such as siRNA or CRISPR-Cas9 components to regulate the gene expression in the cells associated with PVR. This method can selectively suppress genes that promote fibrotic processes, offering a new and innovative treatment option to control PVR.

Although preclinical studies have shown the promise of nanotherapies in treating PVR, the process of translating these findings into clinical practice encounters many obstacles. These tasks involve guaranteeing the safety and compatibility of NPs, refining delivery techniques to bypass the blood–retinal barrier, and building efficient manufacturing procedures on a wide scale. Continued research and clinical trials are essential to tackle these issues and advance the use of nanotherapeutics for the management of PVR.

### 7.1. Blood–Ocular Barriers

Accessing the vitreoretinal microenvironment is uncertain due to the presence of blood–ocular barriers that must be overcome. Developing nanotherapies that can effectively target and treat proliferative membranes on the vitreoretinal surface is currently not viable or, at the absolute least, extremely problematic. Nanoparticles should cross dynamic and static blood–ocular barriers and have therapeutic effects for a certain length of time as well as sustained durations [49]. Therefore, appropriate minimally invasive delivery systems for nanotherapies have to be designed and projected from a pharmacological point of view. In addition, once the correct place contacting the fibrous membranes is located, drugs integrating hydrogels with polymeric nanoparticles, dendrimers, micelles, and liposomes should be released to—hopefully—exert an effective and proper outcome in order to reduce the rate of inflammation and the density of the fibrotic tractions [50].

### 7.2. Safety and Efficacy

Successful nanotherapies aimed at the vitreoretinal interface should be sterile, safe, effective, and efficient. Translating drug-delivery systems for nanotherapies has several critical issues such as the shape, size, functionality, and targetability. Although innovative methods of production such as microfluidizer technology, membrane extrusion, and fluid technology can upscale manufacturing, problems remain with the large-scale generation of functional nanotherapies [51]. Moreover, therapeutic agents should remain biologically active after their delivery to targeted tissues, but this point remains challenging, especially for protein/polypeptide-based therapeutic nanotechnologies. Nanomedicine architecture is an essential aspect of the development process. Reducing toxicity and enhancing efficacy are crucial variables [52]. Tissue engineering and future nanotechnology should be developed to ensure drug-delivery systems with low toxicity as well as long-term stability to rule out the recurrence of several applications. Detailed, extensive, and long-term studies should focus on biocompatibility and the ocular toxicity’s biochemical, ophthalmological, and immunological aspects. Of course, in vitro and in vivo controlled toxicological assessments and human clinical trials have to be carried out before a worldwide release onto the market.

### 7.3. Gene Therapy

Over the last few years, gene therapy has rapidly evolved and many non-viral vectors have been developed that are being investigated for their cytocompatibility, significantly reducing immunogenicity. Non-viral vectors can be formed as part of nanoparticle complexes and can properly deliver genes to specific cell systems. For instance, the innovative use of polyethyleneimine (PEI) is efficient for gene transfer without affecting the retinal or vitreous cell viability or morphology. Moreover, in some studies, the delivery of decorin gene therapy employing PEI nanotechnologies reduced TGF-β-induced fibrosis in in vitro models [53]. This new approach might give birth to a broader use of delivering gene therapies or other therapeutic nanoagents toward distinct parts of the ocular tissues [54]. A recent study reported that polyethylene amine-conjugated gold nanotherapies displayed substantial BMP7 gene delivery into rabbit eye cells in vivo [55]. In addition, it inhibited fibrosis, suggesting that this innovative approach could be versatile in therapeutic applications [56]. However, investigations into an effective treatment for retinal fibrosis and a rapid reduction in PVR formation remain to be explored.

### 7.4. Precision Nanotechnologies

Tailored nanotherapies and precision nanotechnologies are the next steps forward for the therapeutic development of drug-delivery systems. Vitreoretinal diseases such as proliferative retinal affections lead to increasing vascular permeability and, consequently, the rate of inflammation [57]. Precision nanomedicine can be created and designed based on the rate of retinal permeability by controlling drug loading, pharmacokinetics, and release within ocular tissues [58]. Although tailored nanotherapies remain in the early stages of development, future applications of nanomedicine as nanotechniques and nanotools will design better therapeutic molecules and drugs to manage retinal abnormalities such as PVR.

## 8. Expert Opinion

It is necessary to solve numerous current limitations to properly utilize nano-based techniques to control proliferative vitreoretinopathy (PVR). Biocompatibility and toxicity issues are fundamental. Although nanomaterials have distinct benefits in medication administration, their compatibility with living organisms and the possibility of causing harm are significant issues that need to be addressed. Thorough investigations are necessary to comprehend the enduring impacts of nanoparticles in the ocular setting and to guarantee that they do not provoke unfavorable responses. It is difficult to achieve ideal medication loading and regulated release patterns. It is crucial to ensure that nanoparticles effectively deliver therapeutic compounds to the intended location, maintaining the desired dosage and duration while avoiding premature disintegration or release. Moreover, the process of creating nanoparticles that have uniform sizes, shapes, and functional characteristics is extremely challenging. A major obstacle is overcoming the challenge of scaling up these technologies for commercial production while ensuring consistent quality and reproducibility. In addition, obtaining permission for nanomedicines is intricate and rigorous, requiring thorough evaluations in preclinical and clinical settings. Traversing regulatory procedures and acquiring clearance from health authorities can be a protracted and expensive process. Achieving the precise delivery of nanoparticles to the exact target tissue within the eye poses a significant challenge. Indiscriminate dispersion can result in decreased effectiveness and possible adverse reactions [59,60,61].

The topic of nanomedicine in the management of PVR has excellent potential and various future possibilities may address the current constraints and further the subject. The development of novel nanomaterials with superior biocompatibility, increased drug-loading capacity, and precise release mechanisms is crucial. Innovations can improve nanoparticle targeting efficiency and therapeutic efficacy in surface modification and functionalization. Developing nanoparticles that can deliver various therapeutic agents, including anti-inflammatory, anti-proliferative, and anti-fibrotic medicines, could offer a holistic treatment approach for PVR [62,63].

Personalized medicine involves customizing nano-based medicines for individual patients based on their unique illness characteristics and genetic profile, potentially enhancing the treatment outcomes. Customized nanomedicine strategies can improve the accuracy and efficiency of PVR management. By incorporating imaging capabilities into nanoparticles, monitoring the medication distribution and therapeutic effects in real-time becomes possible. This has the potential to expedite prompt modifications to treatments and enhance the overall administration of the disease. It is essential to carry out carefully planned clinical trials to assess the safety and effectiveness of nano-based therapeutics in bigger groups of patients. In addition, gathering empirical data after approval can yield valuable insights into the long-term results and contribute to future enhancements.

## 9. Conclusions

Proliferative vitreoretinopathy (PVR) poses a substantial difficulty in the field of ophthalmology because of its intricate development and the constraints of existing surgical interventions. Although there have been improvements in vitreoretinal surgery such as the use of minimally invasive procedures, the rates of recurrence and complications remain significant. Therefore, it is necessary to investigate alternate treatments. Nanotherapies represent an innovative method to treat proliferative vitreoretinopathy (PVR), providing a hopeful alternative to traditional vitreoretinal surgery. Nanotherapies utilizing the distinctive characteristics of nanoparticles can focus on the particular cellular and molecular pathways associated with PVR. This has the potential to decrease fibrosis and inflammation while minimizing the occurrence of adverse effects. Preclinical investigations have demonstrated the potential effectiveness of various pharmacological treatments, including methotrexate, daunomycin, corticosteroids, retinoic acid, and anti-VEGF drugs. Nevertheless, the complete establishment of their therapeutic efficacy and safety in treating PVR has yet to be determined.

The complexities involved in creating efficient nanotherapies for PVR are manifold. Successfully delivering therapeutic drugs to the vitreoretinal interface, despite the challenges posed by blood–ocular barriers, is a crucial obstacle to overcome. It is crucial for the success of these medicines to maintain stability, biocompatibility, and continuous release inside the ocular environment. Moreover, the process of producing nanotherapeutic agents on a large scale and ensuring their standardization presents considerable obstacles that must be resolved.

Future research in monotherapy should prioritize improving drug-delivery systems to increase precision in targeting and effectiveness in treatment. Gene therapy advancements employing non-viral vectors and nanoparticle complexes show potential for decreasing fibrosis and enhancing retinal repair. Precision nanomedicine, customized to the individual pathological circumstances of the retina, is an advanced method that can potentially completely transform the treatment of PVR.

In the near future, researchers should better classify PVR phenomena by pigeonholing subsets of retinal fibrotic reactions to develop tailored treatments for each specific molecular subset of PVR. The goal is to adopt the surgical approach in fewer cases and to apply effective nanotherapies for most PVR cases to preserve anatomical retinal tissue and reduce the loss of visual function.

Ultimately, thorough and well-planned clinical studies are crucial to confirm the effectiveness and safety of nanotherapies in revolutionizing the treatment of proliferative vitreoretinopathy. To create minimally invasive and successful treatments that can potentially replace or supplement routine vitreoretinal surgery, it is essential to continue to invest in nanotechnology and promote interdisciplinary collaboration. The successful integration of modern nanotechnological methods holds the key to enhancing patient outcomes and quality of life in managing PVR in the future.

## Figures and Tables

**Table 1:** Nano-based drugs for the treatment of proliferative vitreoretinopathy.

Therapy	Nanoformulation	Molecule	Action	Reference
Methotrexate	Intravitreal injections and intraoperative infusions	Folate antagonist	Decrease in RPE cell proliferation and migration, inducing apoptosis and avoiding photoreceptor toxicity	Schulz et al. [29]
Daunomycin	Intraoperative injection	Anthracycline	Inhibition of cell proliferation and migration	Wiedemann et al. [30]
Corticosteroids	Intravitreal triamcinolone acetonide	Steroids	Anti-inflammatory and anti-proliferative properties, various administration methods, and lack of retinal toxicity evidence	Ahmadieh et al. [31], Munir et al. [32]
Retinoic acid	Low-dose oral isotretinoin	Metabolite of vitamin A1	Inhibition of RPE cell growth	Chang et al. [33]
5-Fluorouracil	Intravitreal infusion	Anti-neoplastic antimetabolite	Decrease in PVR growth	Asaria et al. [34]
Mitomycin C	Intravitreal infusion	Anti-tumor agent	Reduction in post-traumatic PVR	Gürelik et al. [35]
Vascular endothelial growth factor Decorin Platelet-derived growth factor receptor MicroRNAs	Intravitreal injection a single intravitreal dose Intravitreal injections Intravitreal administration	Angiogenic factor Proteoglycan Tyrosine kinase receptor RNA micromolecules	Reduction in the bioactivity of vitreous and PVR formation Inhibition of TGF-β and extracellular matrix synthesis Reduction in RPE and glial cell proliferation Inhibition of the RPE–mesenchymal transition	Zhao et al. [36] Abdullatif et al. [37] Zheng et al. [38] Cui et al. [39]
